# Loop-mediated isothermal amplification (LAMP) assay—A rapid detection tool for identifying red fox *(Vulpes vulpes)* DNA in the carcasses of harbour porpoises *(Phocoena phocoena)*

**DOI:** 10.1371/journal.pone.0184349

**Published:** 2017-09-01

**Authors:** Teresa Heers, Abbo van Neer, André Becker, Miguel Luca Grilo, Ursula Siebert, Amir Abdulmawjood

**Affiliations:** 1 Institute for Food Quality and Food Safety, Research Center for Emerging Infections and Zoonoses (RIZ), University of Veterinary Medicine Hannover, Foundation, Hannover, Germany; 2 Institute for Terrestrial and Aquatic Wildlife Research, University of Veterinary Medicine Hannover, Foundation, Hannover, Germany; New York University, UNITED STATES

## Abstract

Carcasses of wild animals are often visited by different scavengers. However, determining which scavenger caused certain types of bite marks is particularly difficult and knowledge thereof is lacking. Therefore, a loop-mediated isothermal amplification (LAMP) assay (target sequence *cytochrome b*) was developed to detect red fox DNA in carcasses of harbour porpoises. The MSwab^™^ method for direct testing without prior DNA isolation was validated. As a detection device, the portable real-time fluorometer Genie^®^ II was used, which yields rapid results and can be used in field studies without huge laboratory equipment. In addition to *in vitro* evaluation and validation, a stranded and scavenged harbour porpoise carcass was successfully examined for red fox DNA residues. The developed LAMP method is a valuable diagnostic tool for confirming presumable red fox bite wounds in harbour porpoises without further DNA isolation steps.

## Introduction

When it comes to forensic studies it is not only important to confirm the actual “offender”, but also to identify and distinguish between predation and postmortem scavenging. Haelters et al. [1] described a case of a live stranded harbour porpoise (*Phocoena phocoena*), which had suffered several injuries. However, these wounds did not meet the typically described injuries of grey seal (*Halichoerus grypus*) attacks, which are known to hunt harbour porpoises [2]. These injuries usually appear as “puncture injuries left by teeth and claws; strips of blubber removed or hanging loose; sometimes muscle partly removed” [1]. Several investigations demonstrated that harbour porpoises are victims of attacks from grey seals [2–6] and in some cases, the DNA of grey seals was isolated from the bite marks of harbour porpoises and confirmed via PCR [2,3]. However, in the case study of Haelters et al. [1], the authors suspected that certain wounds were caused by a red fox scavenging the animal. Unfortunately, it was not possible to prove this assumption because a PCR yielded negative results. Similar problems occurred on harbour porpoises collected through the stranding network of Schleswig-Holstein, Germany where some wound patterns could not be assigned properly. In this context, IJsseldijk and Geelhoed [7] published video recordings showing a red fox actually fed on the carcass of a harbour porpoise. However, red fox DNA detection within the wounds was not performed. Due to the fact that the injuries inflicted by grey seals and red foxes can be confused (“fat and muscle tissues removed; straight wound margin; bite wound in skin and bones” (A. van Neer, pers. comm., March 2017)), for aquatic wildlife researcher it is of great importance to know if the bite marks are related to a grey seal or a red fox. In these cases molecular identification using LAMP (loop-mediated isothermal amplification) assays can be helpful for verification where the predator species identification is only based on bite marks.

The approach of the present study was therefore to establish a novel DNA detection method that is easy to perform, sound, fast and which can be used during forensic field studies without heavy laboratory equipment. Whereas PCR based methods are time-consuming and special equipment is needed, the LAMP assay is a highly specific method which initially used four different primers [8]. Nagamine et al. [9] optimised the LAMP method to include six different primer pairs with two additional loop primers, which ultimately led to an acceleration of the reaction and markedly increased the sensitivity [9]. With the six designed primers a number of eight distinct sequences can be recognised. Moreover, the LAMP method can be performed at a constant temperature and will be complete after 30 minutes. A portable real-time isothermal fluorometer Genie^®^ II (Optigene, United Kingdom) can be used as a detection device during field studies to facilitate and accelerate reliable results.

The LAMP method is already validated for various topics in many research areas. In the field of food safety, bacterial pathogens [10] and fungal contaminants [11] were detected and in case of urgent human health issues, evidence of emerging pathogens like tuberculosis [12] or zika virus disease [13] were possible. Furthermore, identifying animal species in meat products, such as ostrich or pork, was conducted with the LAMP technology [14,15].

To save even more time and make the system more practical, a swab method, described by Abdulmawjood et al. [14], was evaluated to determine the DNA without a previous DNA isolation step.

In the present study, a target sequence of the *cytochrome b* gene of red fox (*Vulpes vulpes)* was selected, primers were designed and the LAMP method was validated *in vitro* and successfully tested on a harbour porpoise which had been stranded on the coastline of Northern Germany and showed possible red fox bite marks. These are typically deep, focal, the blubber being penetrated with bleeding occurring in the underlying tissue [1]. Applying this method it was possible to identify the red fox as a scavenger by directly testing swab samples without previous DNA isolation.

## Materials and methods

### Design of LAMP primer set

**Fig 1:**
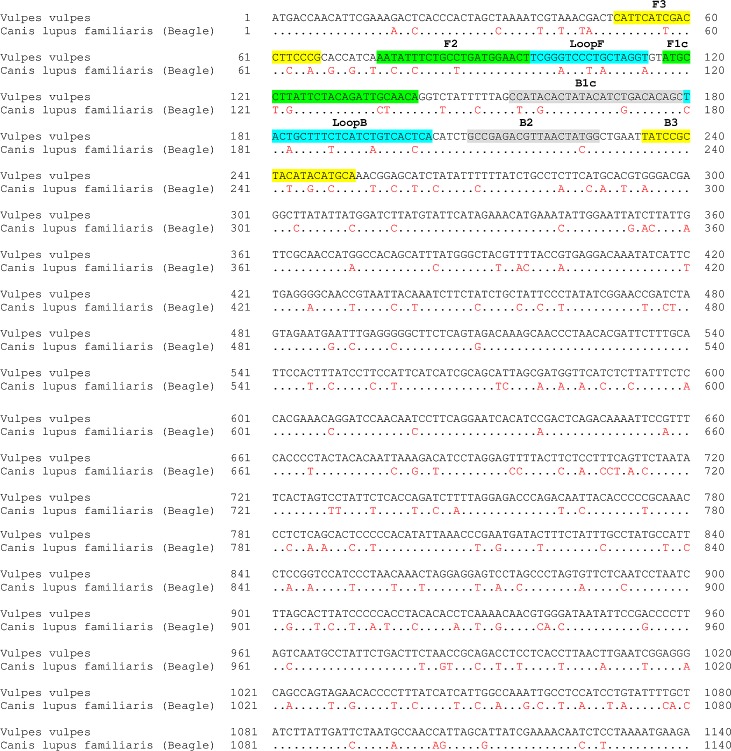
Alignment of the *cytochrome b* sequences of red fox (*Vulpes vulpes*) (accession number AM181037.1) and dog (*Canis lupus familiaris* (Beagle)) (accession number AY729880.1). The dots represent the same base pair, while the red letters show different base pairs. The positions of the LAMP primers for the red fox are colour-coded.

Six specific LAMP primers (forward outer primer VV-CY-F3, backward outer primer VV-CY-B3, forward inner primer VV-CY-FIP, backward inner primer VV-CY-BIP, forward loop primer VV-CY-LoopF and backward loop primer VV-CY-LoopB) were designed using PrimerExplorer V4 (Eiken Chemical Co., Japan). A comparison of the two *cytochrome b* sequences of red fox and dog was performed, using the Basic Local Alignment Search Tool (BLAST) of the National Center for Biotechnology Information (NCBI) (Maryland, USA). For the breed beagle (*Canis lupus familiaris*), which was considered as a model species, the accession number AY729880.1 and for the red fox (*Vulpes vulpes*) the accession number AM181037.1 were used. The alignment of the *cytochrome b* sequences of beagle and red fox resulted in the gene sequences of both animals being 84% identical (Fig 1). The homology for the fox amplicon against the canine amplicon was 85%. The differences in the *cytochrome b* nucleic acid sequence of red foxes and dogs were considered in the selection of a species-specific primer set. All primers were synthesised by Eurofins Genomics (Ebersberg, Germany). A detailed list of the primer sequences is given in Table 1.

**Table 1:** Sequences of the six specific primers for detecting red fox DNA.

Name	Sequence	Length	Melting temperature
VV-CY-F3	5‘-CATTCATCGACCTTCCCG-‘3	18	56.0
VV-CY-B3	5‘-TGCATGTATGTAGCGGATA-‘3	19	52.4
VV-CY-FIP (F1c+F2)	5‘-TGTTGCAATCTGTAGAATAAGGCAT AATATTTCTGCCTGATGGAACT-‘3	47	70.3
VV-CY-BIP (B1c+B2)	5‘-CCATACACTATACATCTGACACAGC CCATAGTTAACGTCTCGGC-‘3	44	74.1
VV-CY-LoopF	5‘-ACCTAGCAGGGACCCGA-‘3	17	57.6
VV-CY-LoopB	5‘-TACTGCTTTCTCATCTGTCACTCA-‘3	24	59.3

### Positive and negative control material

The DNA was isolated from muscle samples of *Vulpes vulpes* (n = 5) which were provided by the Institute for Terrestrial and Aquatic Wildlife Research, University of Veterinary Medicine Hannover, Foundation. The muscle samples of *Canis lupus familiaris* were obtained from the Institute of Pathology, University of Veterinary Medicine Hannover, Foundation. The red fox samples came from the North Sea Coast off Germany (region Eiderstedt). As there are numerous dog breeds in Germany, which can be considered as scavengers, different dog breeds and crossbreeds (n = 19) as representative examples for the variety of breeds in Germany, were investigated (S1 Table). All samples were thoroughly dissected by the staff members of the institutes named above.

### MSwab^™^ method

The MSwab^™^ system (Copan Italia S.p.A., Brescia, Italy) was used to extract DNA without previous DNA purification techniques. In this quick and simple method a flocked swab was stroked across the muscle tissue and subsequently submerged in 1 ml of MSwab^™^ medium, containing Tris HCl, EDTA, TRIS Base, Dimethyl Sulfoxide (DMSO), Bovine Serum Albumin and distilled water. Finally, the reaction tube was shaken by hand and 8 μl was used directly in the LAMP reaction.

### DNA isolation

The DNA extraction of the muscle samples was performed with the commercial DNeasy^®^ blood and tissue kit (Qiagen, Darmstadt, Germany), in accordance with the manufacturer’s instructions, using the protocol “Purification of Total DNA from Animal Tissues (Spin-Column Protocol)”. In brief, 25 mg of the red fox or dog muscle was mixed with 200 μl lysis buffer and 20 μl proteinase K and incubated. After lysis, the DNA was processed according to the manufacturer’s instructions. All positive and negative DNA controls used during the validation and application of the LAMP assay were isolated from muscle samples.

To increase the sensitivity of the MSwab^™^ method mentioned above, the DNA from the MSwab^™^ medium was also extracted using the protocol “Purification of Total DNA from Animal Blood or Cells (Spin-Column Protocol)”. Subsequently, the DNA concentration was measured with the spectrophotometer NanoDrop2000c (Thermo Fisher Scientific, Massachusetts, USA).

### LAMP assay

The loop-mediated isothermal amplification assay was carried out with a real-time fluorometer (Genie^®^ II, Optigene, United Kingdom). The Genie^®^ II is a battery-powered and portable device with two heating blocks. In each heating block eight samples can be investigated simultaneously so that a total number of sixteen samples can be amplified at any one time. The device provides the opportunity to observe the amplification process in real-time on the screen and the analysis will be completed after 30 min.

In accordance with the manufacturer’s instructions, for each sample, 15 μl Isothermal Master Mix ISO-001 (Optigene, United Kingdom) was used with 0.4 μM of the F3-Primer and the B3-Primer. Furthermore, 0.8 μM of the LoopF-Primer and the LoopB-Primer and 1.6 μM of the FIP-Primer and BIP-Primer were admixed and 8 μl of sample DNA were added so that the total reaction volume was 30 μl.

Genie^®^ II works under an isothermal amplification temperature and can be programed for different temperatures. For the specific primer set the optimum temperature was determined as 65°C with detection duration of approximately twenty minutes. The subsequent melting process (in Genie^®^ II called annealing process) starts with 98°C and ends with a temperature of 80°C with a ramp rate of 0.05°C/sec.

### Limit of detection

To determine the limit of detection of the LAMP reaction, a serial dilution with DNA concentrations from 1.45E+04 pg/μl to 1.45E-05 pg/μl was prepared using red fox DNA, isolated from muscle tissue, and AE buffer (10 mM Tris-Cl, 0.5 mM EDTA; pH 9.0). The limit of detection was calculated based on a six-fold verification of the DNA serial dilution.

### Sensitivity and specificity

The sensitivity is the proportion of true positive red fox samples that are correctly identified by the LAMP assay. The specificity is the proportion of true negative dog samples that are correctly identified by the LAMP assay [16]. To verify whether the primer set amplifies red fox DNA correctly as positive, muscle samples of five different animals were tested using the MSwab^™^ protocol. Three swab samples were taken from each thigh muscle sample. The specificity was determined to rule out that the specific primers amplify canine DNA. In each case, three swab samples of 19 muscle samples from different dogs were also tested with the MSwab^™^ method. Each swab was tested directly, without further DNA isolation, with the LAMP assay.

### Spiking experiments

To verify whether the LAMP reaction was working properly under simulated *in vivo* conditions, a skin preparation (thickness 2 cm) of a harbour porpoise was manually incised with a scalpel (approx. 0.5 cm wide and 1 cm deep), imitating red fox bite marks. Subsequently, 10 μl of the isolated red fox DNA from one fold (mean DNA concentration 1.45E+04 pg/μl) to eight fold dilution (mean DNA concentration 1.45E-03 pg/μl) was pipetted thoroughly in the imitated bite mark lesions. After 10 minutes, the tissue was swabbed and the samples were amplified directly using LAMP without prior DNA isolation. The spiking procedure was repeated in triplicate with three independent serial dilutions.

### Necropsy and application of the LAMP assay on a stranded harbour porpoise

**Fig 2:**
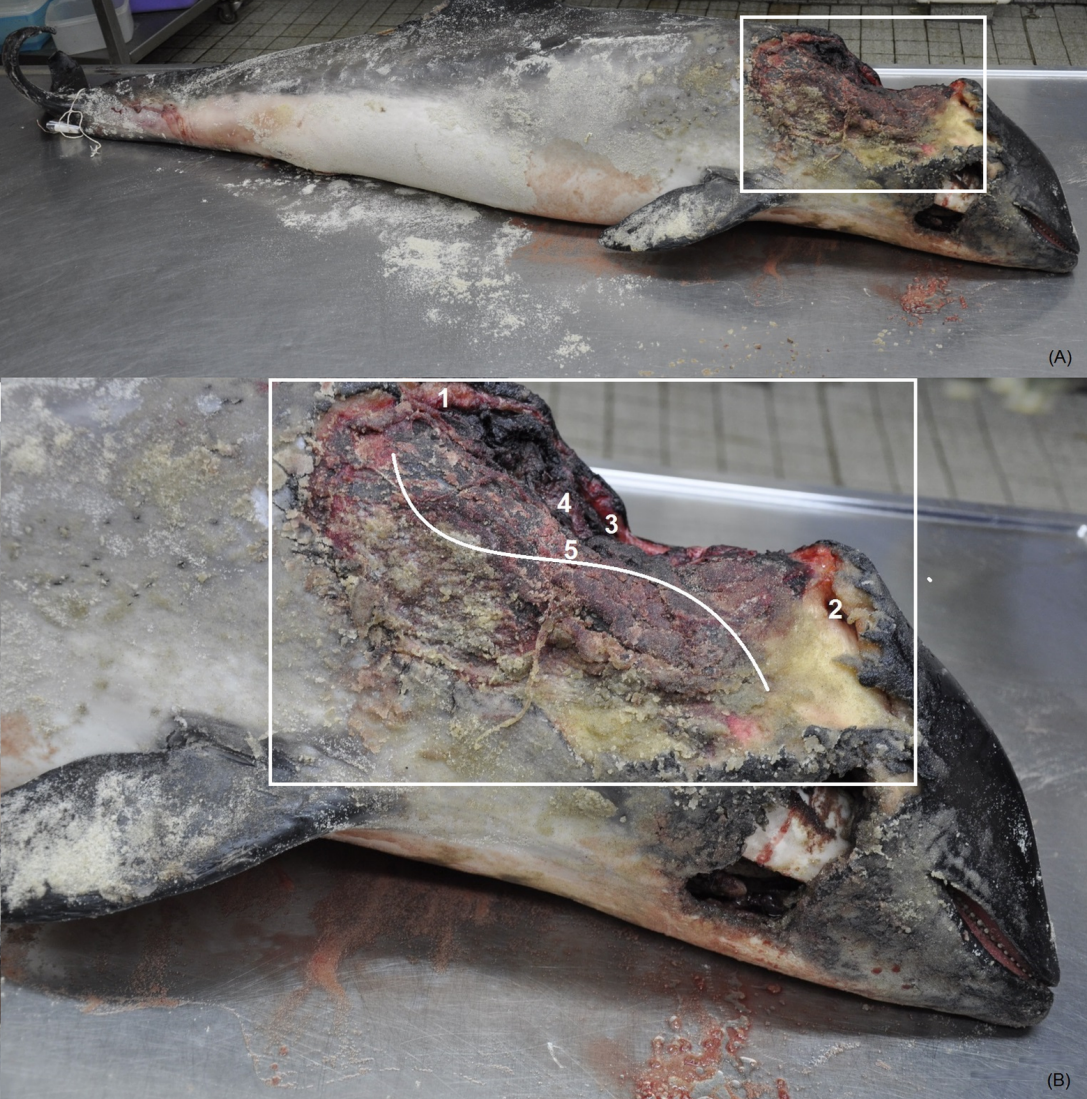
Application of the LAMP assay on a stranded harbour porpoise. (A) Carcass of the stranded harbour porpoise. (B) Sampling locations of the different MSwab^™^ swabs (1–5). Locations were selected at random.

One harbour porpoise carcass (Fig 2A), which was presumably scavenged by a red fox (significant red fox related tracks were observed around the carcass) was collected from the coastline of Northern Germany (Sylt) in October 2016 and transported to the Institute for Terrestrial and Aquatic Wildlife Research in Büsum. The carcass was stored frozen (-20°C) until the necropsy in December 2016. Before sampling, the carcass was thawed at room temperature for three days. During the necropsy, several sterile MSwab^™^ samples were taken from one large wound lesion. Prior to sampling, the dissecting table was treated with the DNA decontamination solution DNA-ExitusPlus^™^ (AppliChem GmbH, Darmstadt, Germany) to eliminate potential previous, undesirable DNA residues. To rule out contamination, the table surface was also tested for red fox DNA after this procedure. After sampling (Fig 2B), the necropsy was performed according to Siebert et al. [17].

The MSwab^™^ samples were analysed by LAMP after the necropsy without prior DNA extraction. Additionally, the DNA within the MSwab^™^ medium was purified and another LAMP reaction was performed to increase the sensitivity of the assay. Concerning the similarity of injuries of the grey seal and the red fox, in both runs the isolated DNA of a grey seal muscle was used as a negative control. Additionally isolated DNA of a dog muscle and isolated DNA of a harbour porpoise muscle were applied as negative control as well.

Finally, the LAMP products of the non-extracted and the extracted DNA were confirmed with gel electrophoresis. Additionally, four other stranded carcasses, including two harbour porpoises, one harbour seal and one grey seal were investigated for red fox DNA, using the developed LAMP assay.

### Gel electrophoresis

To detect the LAMP amplicons, a gel electrophoresis was conducted with 8 μl of the LAMP products, using 2% agarose gel (peqGOLD Universal agarose gel, PeQlab, Erlangen, Germany) and Tris-Borat-EDTA-buffer (pH 8.4). A DNA marker for 50 bp-2 kb (Biozym, Hessisch Oldendorf, Germany) was used.

## Results

### Limit of detection, sensitivity and specificity

**Fig 3:**
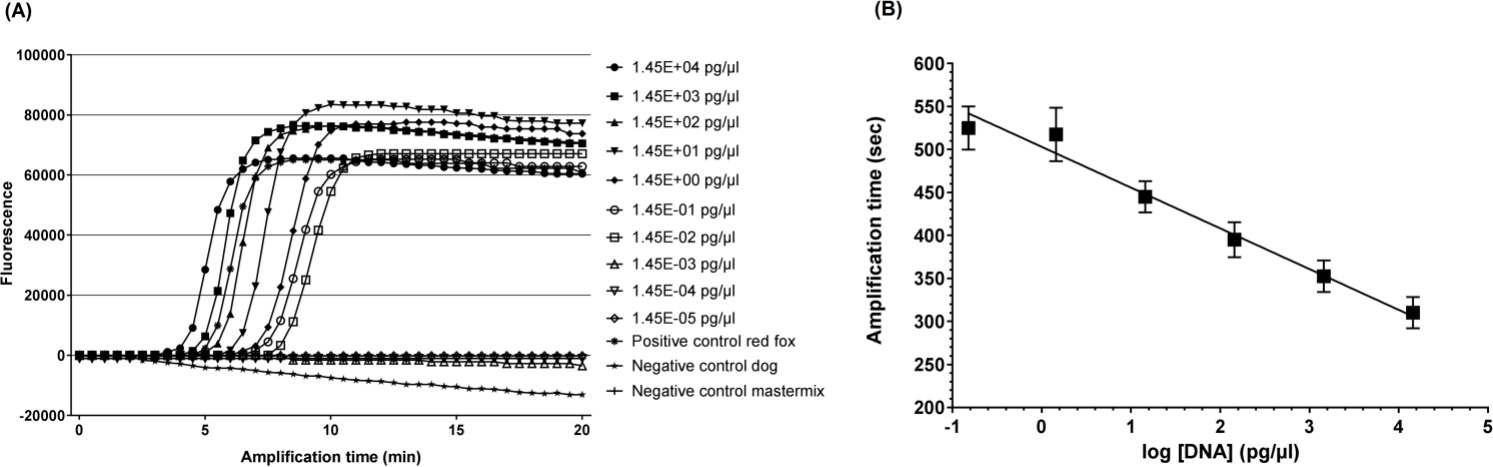
Limit of detection of the LAMP assay. (A) Typical amplification curves of ten-fold serially diluted red fox DNA. (B) The standard curve was generated from a dilution series of the DNA of *Vulpes vulpes* by plotting the amplification time versus the log of the DNA concentration. At concentrations below 1.45E-02 pg/μl the data falls off. The correlation is a linear response.

Typical amplification curves of the diluted red fox DNA, using the real-time fluorometer Genie^®^ II, are shown in Fig 3A. The detection probability was 100% up to a DNA concentration of 1.45E-02 pg/μl with a detection time of 09:02 min (± 00:20 min) (Fig 3). The detection probability dropped to 83% at a DNA concentration of 1.45E-03 pg/μl (mean detection time 13:12 minutes) (S2 Table). The mean of the annealing temperature was 83.1°C (± 0.1°C).

For the sensitivity, five muscle samples of different red foxes were tested directly (without DNA isolation) with the MSwab^™^ method. With the specific primer set each of the samples gave a correctly positive amplification. The mean of the detection time was 13:09 min (± 03:58 min) and the mean temperature of the annealing curve was 82.3°C (± 0.2°C). For the specificity a number of 57 MSwab^™^ samples of 19 different dog muscles were tested without further DNA isolation step. All samples were negative as tested.

### Spiking experiments

The detection of the DNA of the first trial was possible up to the concentration 1.45E+02 pg/μl DNA/bite mark. The mean detection time in that case was 18:07 min (±01:07 min) (Table 2). The minimum DNA concentration of second trial which could be detected was 1.29E+03 pg/μl DNA/bite mark and the mean detection time was 18:22 min (± 00:37 min). The third trial showed a detection time of 18:37 min (± 00:37 min) with a minimum concentration of 1.6E+03 pg/μl. None of the swab sample was extracted with a further DNA isolation step.

**Table 2:** Spiking experiments with the three serial dilutions of the DNA of *Vulpes vulpes*.

Experiment number	Dilution step	DNA concentration (pg/μl)	Detection time (mm:ss)			Mean of the amplification (SD ±)	Detection probability (%)
			run 1	run 2	run 3		
1	10^−1^	1.45E+04	16:00	12:15	13:15	13:50 (01:35)	100
	10^−2^	1.45E+03	19:15	15:00	15:00	16:25 (02:00)	100
	10^−3^	1.45E+02	ND^a^	17:00	19:15	18:07 (01:07)	66.67
	10^−4^	1.45E+01	ND	ND	ND		
2	10^−1^	1.29E+04	12:00	10:30	15:00	12:30 (01:52)	100
	10^−2^	1.29E+03	17:45	ND	19:00	18:22 (00:37)	66.67
	10^−3^	1.29E+02	ND	ND	ND		
3	10^−1^	1.6E+04	18:15	14:30	17:30	16:45 (01:37)	100
	10^−2^	1.6E+03	18:00	19:15	ND	18:37 (00:37)	66.67
	10^−3^	1.6E+02	ND	ND	ND		

The results represent the detection time of non-extracted DNA.

^a^ Not Detected.

### Necropsy

The female adult harbour porpoise, with a length of 159 cm, presented a large tissue defect in the right dorso-lateral side of the cranial part of the body. The defect extended from the caudal side of the blowhole to the area dorsal to the right front flipper, extending from the dorsum of the animal to the level of the right ramus of the mandible. The majority of skin, blubber and muscular tissue in the wound area was missing. The wound’s margins in its left and cranial right side were regular, whereas the other edges were irregular. In several sites of the wound margin it was possible to detect triangular-shaped edges where the margin had a regular surface.

### Application of the LAMP assay on a stranded harbour porpoise

Initially, a total number of eight swabs were tested without DNA isolation (Table 3). The three control swab samples of the dissecting table were all negative. Swabs 1, 2 and 3 did not show an amplification signal with the specific primer. However, the swab samples 4 and 5 were positive after 16:30 min and 15:45 min, respectively. The positive control was already detected after 5:30 min. All negative controls were not amplified with the selected primer set.

**Table 3:** LAMP results of the first stranded harbour porpoise investigated for red fox DNA residues.

Sample	Without DNA isolation		With DNA isolation	
	Detection time (mm:ss)	Anneal Derivative (°C)	Detection time (mm:ss)	Anneal Derivative (°C)
MSwab^™^ Dissecting Table 1	ND^b^	ND	ND	ND
MSwab^™^ Dissecting Table 2	ND	ND	ND	ND
MSwab^™^ Dissecting Table 3	ND	ND	ND	ND
MSwab^™^ 1	ND	ND	11:15	82.8
MSwab^™^ 2	ND	ND	15:00	81.2
MSwab^™^ 3	ND	ND	10:00	82.9
MSwab^™^ 4	16:30	81.9	10:30	82.8
MSwab^™^ 5	15:45	82.1	12:15	82.9
Negative control Harbour porpoise^a^	ND	ND	ND	ND
Negative control Grey Seal^a^	ND	ND	ND	ND
Negative control Dog^a^	ND	ND	ND	ND
Positive control Red fox^a^	5:30	82.8	5:15	82.6

^a^ Isolated DNA from muscle samples

^b^ Not Detected.

**Fig 4:**
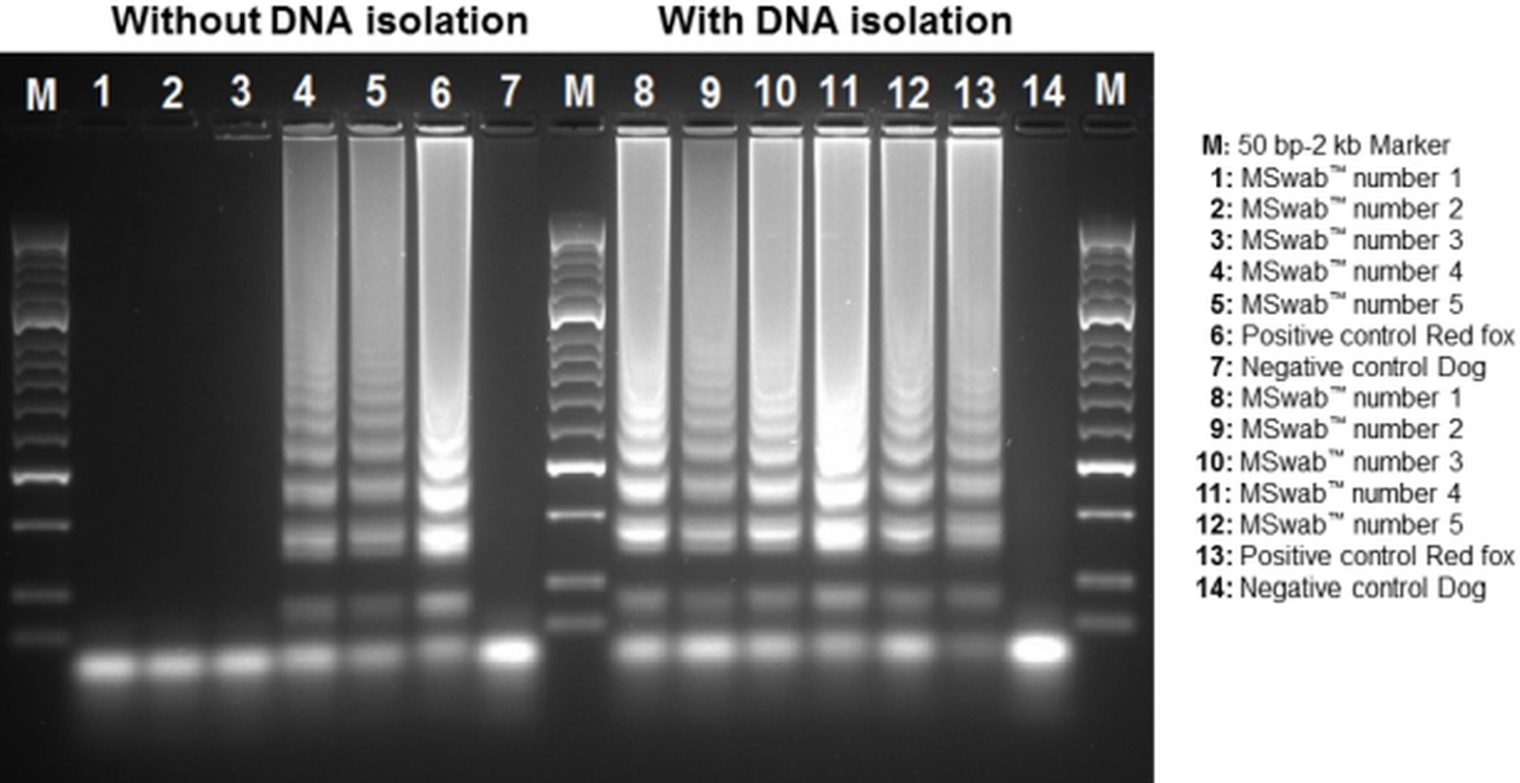
Application of the LAMP assay on a stranded harbour porpoise. Gel electrophoresis of all LAMP products of the stranded harbour porpoise. Directly tested MSwab^™^ samples (without DNA isolation) and DNA isolation of the MSwab^™^ medium using DNeasy^®^ blood and tissue kit.

Besides the direct sample testing, DNA was isolated from the Mswab^™^ samples and subsequently tested with the LAMP assay (Table 3). With this additional purification step, the DNA detection was possible in all bite mark samples. The results of both direct and isolated samples were confirmed by gel electrophoresis (Fig 4).

As expected, the detection time of the directly positive 4 and 5 swab samples was longer (swab no. 4 6:00 min longer and swab no. 5 3:30 min longer) than the detection time of the isolated DNA.

The other two investigated harbour porpoises, the harbour seal and the grey seal were negative for red fox DNA residues.

## Discussion

The results of this project show that it is possible to detect red fox DNA residues in the wounds of a harbour porpoise, using the LAMP technology. In this case it was presumed to find red fox DNA in the bite marks, as significant tracks of a red fox were viewed around the stranded carcass. In the other four cases, of negatively tested marine mammals it was not known if a red fox visited the carcasses. These results indicate that no red fox was feeding on those carcasses or the DNA of the red fox in the bite marks was degraded.

On a Dutch coastline the red fox was identified as a scavenger with video-recording proof [7]. However, molecular biological identification of a predator can confirm an observation or identity of the predator without visual proof [18]. Besides that, the predation itself cannot be observed regularly anyhow, so a test based on DNA analysis is needed.

Confirmation of DNA residues in wounds is not an easy task and brings challenges for the sampling and analysis process. Although previous studies succeeded in isolating predator DNA of grey seals from wounds of harbour porpoises via PCR [3,5], this was not successful for detecting red fox DNA [1]. This shows that PCR analyses might not always be the method of choice when the objective is to detect scavenger DNA residues. A good alternative to PCR is the loop-mediated isothermal amplification assay. This relatively modern method is reported to be rapid and easy to apply [8]. LAMP is a highly specific method with particular high target selectivity, due to the fact that six primers bind to eight specific target sequences [9], this being the main reason for using the LAMP technology in the present study. Additionally, the use of Genie^®^ II as a portable detection device offers the possibility of performing analysis during field studies in areas where there is no easy access to laboratories. As DNA within wounds is very sensitive, this also has the advantage of avoiding degradation or contamination during the transportation process [19]. Another benefit of the LAMP assay is its greater sensitivity compared to the PCR [20,21]. This plays an important role when working with saliva samples, as they contain relatively low quantities of DNA, but offer a good opportunity for solving forensic questions in wildlife areas [22]. Usually, the DNA must be isolated from a sample before initiating a PCR. With the LAMP assay in combination with a swab method, no further DNA extraction step is necessary and thus the swab samples can be tested directly and user-friendly [23].

Alongside red foxes, dogs are known to be potential scavengers of harbour porpoises and in some cases even the most abundant ones in Australia [1,4,5,24]. As there is a close relation between the mitochondrial DNA of red foxes and dogs, the *cytochrome b* sequence of dogs was considered and successfully ruled out during the primer design and amplification process.

The results of our study confirm that taking DNA samples is an effective method to clarify forensic questions in the field of wildlife research, since we succeeded in detecting the DNA of *Vulpes vulpes* in the bite lesion of a stranded harbour porpoise. In various wildlife projects, researchers were able to show that saliva samples can be used to identify different predators. For example, DNA of coyotes [19,25] or wolves [26] was detected in sheep carcasses. A substantial problem is that DNA originating from saliva is subject to different types of degradation, like enzymes, bacteria or weather conditions [27,28]. Furthermore, Pääbo et al. [28] pointed out that low temperatures and dry conditions lead to a slower DNA degradation. Moreover, due to sea water there may be a dilution of the DNA.

Besides using the LAMP method, this may explain the unsuccessful evidence of red fox DNA via PCR in the project of Haelters et al. [1] and the negative results of the other four tested stranded marine mammals in this study in comparison to our positive results.

In this context, it has to be pointed out that in our study only two out of the five directly tested swab samples of the harbour porpoise carcass showed a positive amplification. This may be the result of a low DNA quantity due to degradation, as after DNA isolation of the swab samples and the associated higher yield or accessibility of DNA all samples were detected as positive.

We recommend to swab over the whole lesion or in the middle of the lesion as these were the locations of the directly positive swabs in our case. It can also be beneficial to take several samples within these locations or increase the amplification time from 20 to 30 minutes. In the performed application of the LAMP assay each sample of the dissecting table yielded negative results for red fox DNA residues. Thus, it is further recommended to take swab samples of the dissecting table as quality assurance and to avoid false positive results.

Although the MSwab^™^ method offers the advantage of direct DNA testing, it was astonishing that the detection limit in the spiking experiment was rather poor (1.45E+02 pg/μl) in comparison to 1.45E-03 pg/μl during the limit of detection testing. This shows that there is a significant DNA quantity lost during sampling because of a dilution in 1 ml MSwab^™^ medium and a huge matrix effect due to inhibitor factors like fat and blood within the wound lesion, making it even more difficult to catch particularly low concentrations of DNA residues within deep wounds. Nevertheless, with the stranded harbour porpoise we were able to demonstrate that the DNA quantity is still sufficient to detect red fox DNA with the MSwab^™^ under simulated *in vivo* conditions.

Fast and specific DNA detection methods will always be a major topic for forensic studies and with the developed LAMP assay it is possible to detect the red fox as the scavenger of marine mammals. The field-use of the Genie^®^ II fluorometer is simple as it is possible to blend the master mix in advance so there is only the need for the MSwab^™^, a pipette and the portable and battery-powered Genie^®^II device on-site. The samples can then be transferred directly into the LAMP assay to identify the scavenger or predator immediately but can also be purified to increase sensitivity.

## Conclusion

The developed LAMP method is a valuable diagnostic tool for wildlife researchers to identify red fox DNA in carcasses of marine mammals. In this study we could successfully confirm that wound lesions, presumably caused by red fox bites, were actually red fox-related. The presented method can be performed on-site, fast and without the need for large laboratory equipment.

## Supporting information

S1 Table Dog breed samples (n = 19) investigated in the present study as negative controls for the exclusivity test. (PDF)

S2 Table Limit of detection of the LAMP assay by using serial dilutions of the DNA of *Vulpes vulpes*. (PDF)

## References

[1] Haelters J, Everaarts E, Bunskeok P, Begeman L, Hinrichs JWJ, Ijsseldijk LL (2016) A Suspected Scavenging Event by Red Foxes (Vulpes vulpes) on a Live, Stranded Harbour Porpoise (Phocoena phocoena). Aquatic Mammals 42: 227–232.

[2] Leopold MF, Begeman L, van Bleijswijk JDL, Ijsseldijk LL, Witte HJ, Gröne A (2014) Exposing the grey seal as a major predator of harbour porpoises. Proceedings of the Royal Society B: Biological Sciences 282. 10.1098/rspb.2014.2429 PMC4262184 25429021

[3] van Bleijswijk JDL, Begeman L, Witte HJ, Ijsseldijk LL, Brasseur S, Gröne A, et al (2014) Detection of grey seal Halichoerus grypus DNA in attack wounds on stranded harbour porpoises Phocoena phocoena. Marine Ecology Progress Series 513: 277–281.

[4] Haelters J, Kerkchof F, Jauniaux T, Degraer S (2012) The Grey Seal (Halichoerus grypus) as a Predator of Harbour Porpioses (Phocoena phocoena)? Aquatic Mammals: 343–353.

[5] Jauniaux T, Garigliany MM, Loos P, Bourgain JL, Bouveroux T, Coignoul F, et al (2014) Bite injuries of grey seals (Halichoerus grypus) on harbour porpoises (Phocoena phocoena). PLoS One 9: e108993 doi: 10.1371/journal.pone.0108993 25461599 10.1371/journal.pone.0108993 PMC4251829

[6] Stringell T, Hill D, Rees D, Rees F, Rees P, Morgan G (2015) Short Note: Predation of Harbour Porpoises (Phocoena phocoena) by Grey Seals (Halichoerus grypus) in Wales. Aquatic Mammals 41: 188–191.

[7] IJsseldijk L, Geelhoed S (2016) Fox scavenging mutilations on dead harbour porpoises (Phocoena phocoena). IMARES Report [LXXX/JJ].

[8] Notomi T, Okayama H, Masubuchi H, Yonekawa T, Watanabe K, Amino N, et al (2000) Loop-mediated isothermal amplification of DNA. Nucleic Acids Res 28: E63 10871386 10.1093/nar/28.12.e63 PMC102748

[9] Nagamine K, Hase T, Notomi T (2002) Accelerated reaction by loop-mediated isothermal amplification using loop primers. Mol Cell Probes 16: 223–229. 12144774 10.1006/mcpr.2002.0415

[10] Sheet OH, Grabowski NT, Klein G, Abdulmawjood A (2016) Development and validation of a loop mediated isothermal amplification (LAMP) assay for the detection of Staphylococcus aureus in bovine mastitis milk samples. Mol Cell Probes 30: 320–325. doi: 10.1016/j.mcp.2016.08.001 27495132 10.1016/j.mcp.2016.08.001

[11] Abd-Elsalam K, Bahkali A, Moslem M, Amin OE, Niessen L (2011) An optimized protocol for DNA extraction from wheat seeds and Loop-Mediated Isothermal Amplification (LAMP) to detect Fusarium graminearum contamination of wheat grain. Int J Mol Sci 12: 3459–3472. doi: 10.3390/ijms12063459 21747688 10.3390/ijms12063459 PMC3131572

[12] Organization WH (2016) The Use of Loop-Mediated Isothermal Amplification (TB-LAMP) for the Diagnosis of Pulmonary Tuberculosis: Policy Guidance. WHO Verlag, World Health Organization, Genf, Schweiz. 27606385

[13] Lee D, Shin Y, Chung S, Hwang KS, Yoon DS, Lee JH (2016) Simple and Highly Sensitive Molecular Diagnosis of Zika Virus by Lateral Flow Assays. Anal Chem 88: 12272–12278. doi: 10.1021/acs.analchem.6b03460 28193014 10.1021/acs.analchem.6b03460

[14] Abdulmawjood A, Grabowski N, Fohler S, Kittler S, Nagengast H, Klein G (2014) Development of loop-mediated isothermal amplification (LAMP) assay for rapid and sensitive identification of ostrich meat. PLoS One 9: e100717 doi: 10.1371/journal.pone.0100717 24963709 10.1371/journal.pone.0100717 PMC4071038

[15] Lee S-Y, Kim M-J, Hong Y, Kim H-Y (2016) Development of a rapid on-site detection method for pork in processed meat products using real-time loop-mediated isothermal amplification. Food Control 66: 53–61.

[16] Altman DG, Bland JM (1994) Diagnostic tests 1: sensitivity and specificity. BMJ 308: 1552 8019315 10.1136/bmj.308.6943.1552 PMC2540489

[17] Siebert U, Wunschmann A, Weiss R, Frank H, Benke H, Frese K (2001) Post-mortem findings in harbour porpoises (Phocoena phocoena) from the German North and Baltic Seas. Journal of Comparative Pathology 124: 102–114. doi: 10.1053/jcpa.2000.0436 11222006 10.1053/jcpa.2000.0436

[18] Mumma MA, Soulliere CE, Mahoney SP, Waits LP (2013) Enhanced understanding of predator-prey relationships using molecular methods to identify predator species, individual and sex. Mol Ecol Resour 14: 100–108. doi: 10.1111/1755-0998.12153 23957886 10.1111/1755-0998.12153

[19] Williams CL, Blejwas K, Johnston JJ, Jaeger MM (2003) A Coyote in Sheep's Clothing: Predator Identification from Saliva. Wildlife Society Bulletin Vol. 31: 926–932.

[20] Parida M, Posadas G, Inoue S, Hasebe F, Morita K (2004) Real-Time Reverse Transcription Loop-Mediated Isothermal Amplification for Rapid Detection of West Nile Virus. Journal of Clinical Microbiology 42: 257–263. doi: 10.1128/JCM.42.1.257-263.2004 14715762 10.1128/JCM.42.1.257-263.2004 PMC321710

[21] Parida M, Horioke K, Ishida H, Dash PK, Saxena P, Jana AM, et al (2005) Rapid detection and differentiation of dengue virus serotypes by a real-time reverse transcription-loop-mediated isothermal amplification assay. J Clin Microbiol 43: 2895–2903. 15956414 10.1128/JCM.43.6.2895-2903.2005 PMC1151941

[22] Harms V, Nowak C, Carl S, Muñoz-Fuentes V (2015) Experimental evaluation of genetic predator identification from saliva traces on wildlife kills. Journal of Mammalogy 96: 138–143.

[23] Kaneko H, Kawana T, Fukushima E, Suzutani T (2007) Tolerance of loop-mediated isothermal amplification to a culture medium and biological substances. J Biochem Biophys Methods 70: 499–501. doi: 10.1016/j.jbbm.2006.08.008 17011631 10.1016/j.jbbm.2006.08.008

[24] Schlacher TA, Weston MA, Lynn D, Schoeman DS, Huijbers CM, Olds AD, et al (2014) Conservation gone to the dogs: when canids rule the beach in small coastal reserves. Biodiversity and Conservation 24: 493–509.

[25] Blejwas K, Williams CL, Shin GT, McCullough DR, Jaeger MM (2006) Salivary DNA Evidence Convicts Breeding Male Coyotes of Killing Sheep. Journal of Wildlife management 70: 1087–1093.

[26] Caniglia R, Fabbri E, Mastrogiuseppe L, Randi E (2013) Who is who? Identification of livestock predators using forensic genetic approaches. Forensic Sci Int Genet 7: 397–404. doi: 10.1016/j.fsigen.2012.11.001 23200859 10.1016/j.fsigen.2012.11.001

[27] Williams CL, Johnston JJ (2004) Using Genetic Analyses to Identify Predators. Sheep & Goat Research Journal 19: 85–88.

[28] Pääbo S, Poinar H, Serre D, Jaenicke-Despres V, Hebler J, Rohland N, et al (2004) Genetic analyses from ancient DNA. Annu Rev Genet 38: 645–679. doi: 10.1146/annurev.genet.37.110801.143214 15568989 10.1146/annurev.genet.37.110801.143214

