# Supplementary material for: Loop-mediated isothermal amplification (LAMP) assay—A rapid detection tool for identifying red fox (Vulpes vulpes) DNA in the carcasses of harbour porpoises (Phocoena phocoena)
Source: PLoS One. 2017 Sep 1;12(9):e0184349. doi: 10.1371/journal.pone.0184349 (PMC5581184; doi:10.1371/journal.pone.0184349)
Supplement: S1 Table — (PDF) [file pone.0184349.s001.pdf]

S1 Table. Dog breed samples (n=19) investigated in the present study as negative controls for the exclusivity test.

Gender	Breed
Male	Springer Spaniel
Castrated female	Schnauzer
Female	Great Dane
Male	Jack Russel Terrier
Male	Eurasier
Castrated male	Crossbreed
Female	Labrador Retriever
Female	Crossbreed
Male	Crossbreed
Male	Cairn Terrier
Female	Yorkshire Terrier
Male	Beagle
Female	French Bulldog
Female	Labrador Retriever
Male	Dogo Argentino
Male	Tibetan Mastiff (Do Khyi)
Female	Beagle
Female	Affenpinscher
Male	Lagotto Romagnolo
