# Supplementary material for: Loop-mediated isothermal amplification (LAMP) assay—A rapid detection tool for identifying red fox (Vulpes vulpes) DNA in the carcasses of harbour porpoises (Phocoena phocoena)
Source: PLoS One. 2017 Sep 1;12(9):e0184349. doi: 10.1371/journal.pone.0184349 (PMC5581184; doi:10.1371/journal.pone.0184349)
Supplement: S2 Table — (PDF) [file pone.0184349.s002.pdf]

S2 Table. Limit of detection of the LAMP assay by using serial dilutions of the DNA of *Vulpes vulpes*.

DNA concentration (pg/µl)	Detection time (mm:ss)						Mean of the amplification (SD ±)	Detection probability (%)
	run 1	run 2	run 3	run 4	run 5	run 6		
1.45E+04	04:45	05:30	05:00	05:00	05:15	05:30	05:10 (00:16)	100
1.45E+03	05:45	05:45	05:45	05:45	05:45	06:30	05:52 (00:16)	100
1.45E+02	06:15	06:30	06:30	06:30	06:30	07:15	06:35 (00:18)	100
1.45E+01	07:15	07:15	07:30	07:15	07:15	08:00	07:25 (00:16)	100
1.45E+00	08:15	08:15	08:30	08:15	08:30	09:30	08:32 (00:26)	100
1.45E-01	08:15	08:30	08:45	08:45	09:00	09:30	08:47 (00:23)	100
1.45E-02	08:45	09:00	09:00	09:00	08:45	09:45	09:02 (00:20)	100
1.45E-03	09:45	18:15	11:00	ND[a]	10:45	16:15	13:12 (03:23)	83
1.45E-04	ND	ND	ND	ND	ND	ND		
1.45E-05	ND	ND	ND	ND	ND	ND		

[a] Not detected.
